# Galacto-oligosaccharide preconditioning improves metabolic activity and engraftment of *Limosilactobacillus reuteri* and stimulates osteoblastogenesis ex vivo

**DOI:** 10.1038/s41598-024-54887-z

**Published:** 2024-02-21

**Authors:** Florac De Bruyn, Nicolas Bonnet, Michaël Baruchet, Magalie Sabatier, Isabelle Breton, Bertrand Bourqui, Ivana Jankovic, Marie-Noëlle Horcajada, Guénolée Prioult

**Affiliations:** 1 Nestlé Research and Development, Nestléstrasse 3, 3510 Konolfingen, Switzerland; 2 Nestlé Institute of Health Sciences, Nestlé Research, EPFL Innovation Park, Lausanne, Switzerland; 3 Nestlé Institute of Health Sciences, Route du Jorat 57, 1000 Lausanne, Switzerland; 4 Nestlé Health Science, Route du Jorat 57, 1000 Lausanne, Switzerland

**Keywords:** *Limosilactobacillus reuteri* DSM 17938, Galacto-oligosaccharides, Probiotic engraftment, Osteoblastogenesis, Microbiology, Gastroenterology

## Abstract

A probiotic-related benefit for the host is inherently linked to metabolic activity and integration in the gut ecosystem. To facilitate these, probiotics are often combined with specific prebiotics in a synbiotic formulation. Here, we propose an approach for improving probiotic metabolic activity and engraftment. By cultivating the probiotic strain in the presence of a specific prebiotic (preconditioning), the bacterial enzymatic machinery is geared towards prebiotic consumption. Today, it is not known if preconditioning constitutes an advantage for the synbiotic concept. Therefore, we assessed the effects galacto-oligosaccharide (GOS) addition and preconditioning on GOS of *Limosilactobacillus reuteri* DSM 17938 on ex vivo colonic metabolic profiles, microbial community dynamics, and osteoblastogenesis. We show that adding GOS and preconditioning *L. reuteri* DSM 17938 act on different scales, yet both increase ex vivo short-chain fatty acid (SCFA) production and engraftment within the microbial community. Furthermore, preconditioned supernatants or SCFA cocktails mirroring these profiles decrease the migration speed of MC3T3-E1 osteoblasts, increase several osteogenic differentiation markers, and stimulate bone mineralization. Thus, our results demonstrate that preconditioning of *L. reuteri* with GOS may represent an incremental advantage for synbiotics by optimizing metabolite production, microbial engraftment, microbiome profile, and increased osteoblastogenesis.

## Introduction

Probiotic activity is the ability to stimulate a proven health benefit to its host. Probiotic strains can achieve this benefit through metabolic activity and engraftment (stable establishment of a bacterial strain in the human gut)^1–5^. To this end, probiotics can be combined with prebiotics. Prebiotics are substrates that are selectively utilized by host microorganisms conferring a health benefit. The rational combination of a probiotic with a specific prebiotic is referred to as a synbiotic. Synbiotics are an effective strategy to improve probiotic survival, engraftment, and performance^6,7^.

Members of *Lactobacillaceae* are common probiotics that are found in a large variety of food products and inhabit the skin and mucosal surfaces of humans^8,9^. *Limosilactobacillus reuteri* (basonym: *Lactobacillus reuteri* Kandler et al*.* 1982, 266^VL^) is commonly isolated as a member of different intestinal microbial ecosystems, including the human gastrointestinal tract (GIT)^10–15^. *Limosilactobacillus reuteri*-associated benefits include reducing infections, improving food tolerance, enhancing absorption of nutrients, minerals, and vitamins, modulating host immune response, and promoting gut mucosal integrity^16–19^. *Limosilactobacillus reuteri* DSM 17938 is one of the most extensively studied strains for remediation of infantile colic^20–22^. Moreover, *L. reuteri* ATCCPTA 6475 is the first probiotic for which a substantial bone health benefit has been demonstrated by reducing bone loss and incident fracture risk^23^. One of the mechanisms of action identified for *L. reuteri*’s effect on bones is through the production of short-chain fatty acids (SCFA), which can both stimulate osteoblast differentiation and inhibit osteoblastogenesis^24^. Indeed, acetate can improve calcium absorption, reduce bone resorption, and increase bone formation depending on its concentration and ratio to other microbial SCFAs like propionate and butyrate^25,26^. Strategies to stimulate the action of *L. reuteri* represent an opportunity for nutritional solutions to improve or maintain bone health.

Galacto-oligosaccharides (GOS) are a family of prebiotics that can be produced from lactose through transgalactosylation by β-galactosidase^27^. Galacto-oligosaccharides and other structurally related oligosaccharides have been shown to improve gastrointestinal comfort, stimulate beneficial gut microbes, and protect infants from gastrointestinal pathogenic bacteria^28,29^. Therefore, infant formulae are sometimes supplemented with GOS to modulate bowel function and gut microbiome composition of bottle-fed babies. Galacto-oligosaccharides also stimulate the growth of lactobacilli and bifidobacteria^30,31^. Different *L. reuteri* strains can utilize GOS, a phenotype which possibly conveys an ecological advantage for these strains in the GIT^32^.

In this paper, we propose an approach that consists of priming the bacterial enzymatic machinery of a probiotic strain for a specific prebiotic by having a structurally similar substrate as a carbon source during production. We will refer to this approach as preconditioning. Preconditioning can result in a competitive advantage for the preconditioned strain when used in a synbiotic formulation containing the said prebiotic. Hence, preconditioning could lead to improved metabolic activity, probiotic engraftment, or other benefits. Preconditioning of *Bifidobacterium lactis* CNCM I-3446 has been shown to enhance early microbial activity in ex vivo colonic simulations before^33^. However, as of today, it is not known if preconditioning does constitute an advantage that could potentially go beyond microbial activity or if it is translatable to other probiotics. Therefore, we assessed the effects of infant formula supplemented with GOS and preconditioning on GOS of *L. reuteri* DSM 17938 on ex vivo metabolic profiles, microbial community dynamics, probiotic engraftment, and ex vivo osteoblastogenesis. To this end, using a colonic incubation model we show that GOS addition and *L. reuteri* DSM 17938 preconditioning act on different scales, yet both increase SCFA production and probiotic engraftment. Further, supernatants from harvested from this model enhance bone formation (osteoblast activity).

## Results

**Figure 1:**
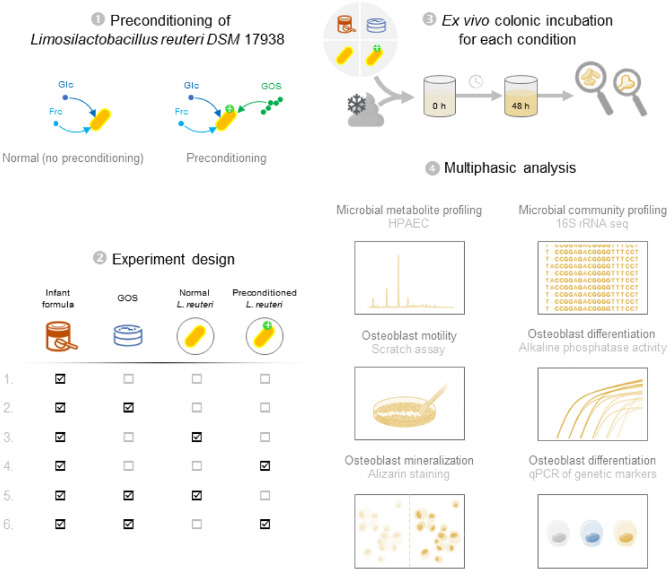
Overview of the experiments and analyses performed. (1) *Limosilactobacillus reuteri* DSM 17938 is produced through the normal production process, in which glucose (Glc) and fructose (Frc) are available as carbon sources. The probiotic strain is also produced through the preconditioning process, in which galacto-oligosaccharides (GOS), glucose (Glc), and fructose (Frc) are available as carbon sources. (2) The six experimental conditions we assessed consist of selective additions of the components galacto-oligosaccharide (GOS), normal (non-preconditioned) *L. reuteri* DSM 17938, and preconditioned *L. reuteri* DSM 17938. The different combinations of these components allow to assess each component individually. Infant formula was added in all cases. (3) Selected component mixes and cryopreserved toddler fecal inoculum were added to controlled ex vivo colonic incubators and sampled at 0 and 48 h of incubation time. Every condition was performed in biological triplicate. (4) Samples were analyzed through a multiphasic pipeline consisting of a microbiological and osteal part.

We present an overview of the experimental setup in Fig. 1.

### Galacto-oligosaccharide addition and preconditioning *Limosilactobacillus reuteri* DSM 17938 modulate ex vivo colonic metabolite profiles

**Figure 2:**
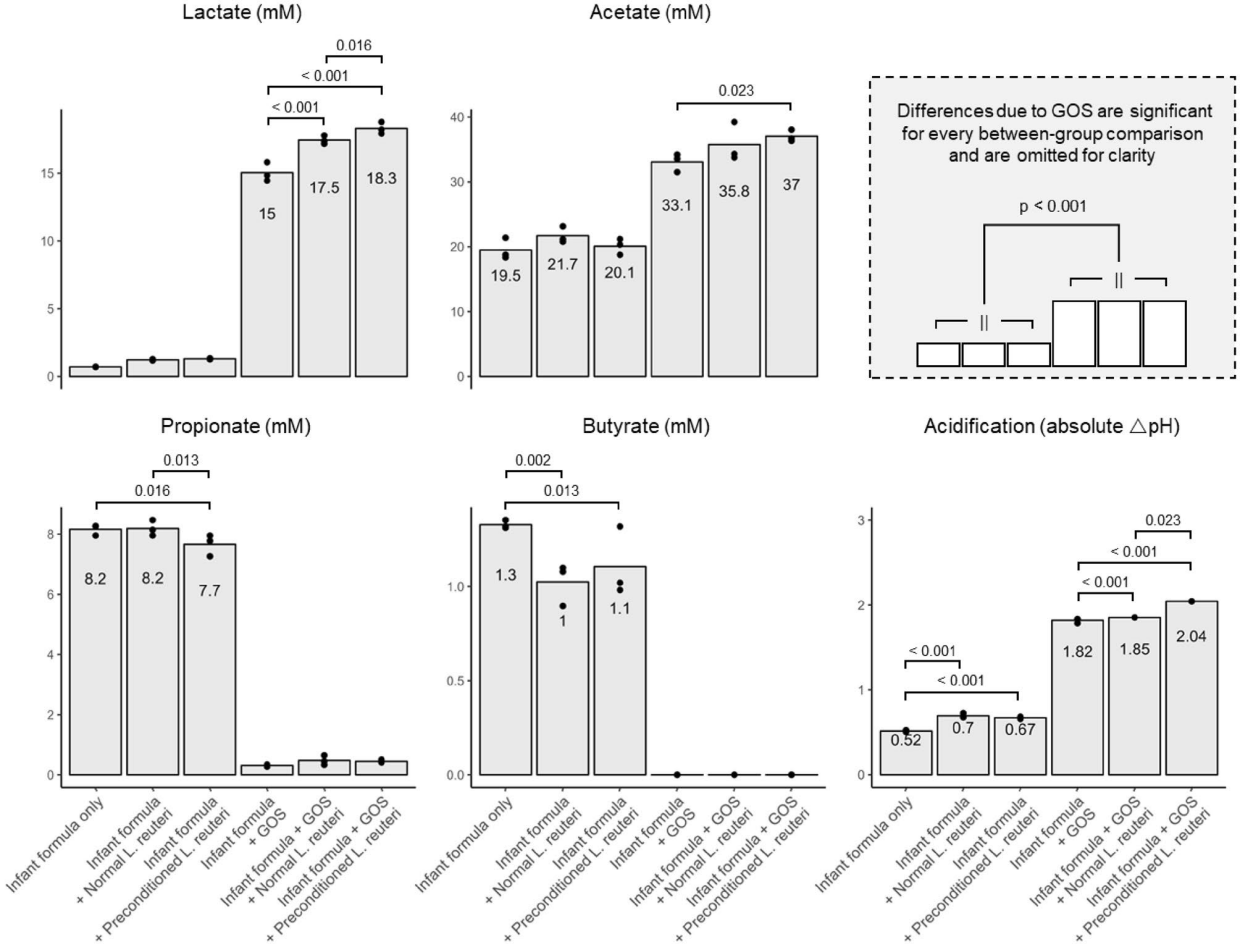
Microbial metabolite profiles after 48 h of ex vivo colonic incubation are affected by GOS and preconditioning. Dots denote individual replicates (n = 3) and bars their average. The effect of galacto-oligosaccharide (GOS) addition is apparent through strong increases in lactate and acetate upon addition. The effect of *Limosilactobacillus reuteri* DSM 17938 preconditioning is apparent to a lesser extent. Lactate and acetate (key metabolites of *L. reuteri* DSM 17938) show upward trends when moving from non-preconditioned to preconditioned *L. reuteri* DSM 17938 conditions. The same trend is observed for acidification. Notably, preconditioning *L. reuteri* DSM 17938 significantly increases lactate concentration and acidification, compared to the non-preconditioned strain. Pairwise p-values are denoted with a bracket. Differences due to GOS addition are significant for all conditions and thus omitted from the plot for clarity.

The metabolite profiles during ex vivo colonic incubation were primarily affected by galacto-oligosaccharide (GOS) addition and secondarily by *Limosilactobacillus reuteri* DSM 17938, both normal and preconditioned (Fig. 2). Adding GOS increased final lactate and acetate concentrations and concomitantly decreased pH. Conversely, omitting GOS resulted in higher concentrations of propionate and butyrate. Differences due to GOS addition were significant for all conditions (p < 0.001). Still, preconditioning effects were apparent in presence of GOS, as concentrations of *L. reuteri* DSM 17938 main end-metabolites (lactate and acetate) were increased for these conditions. When adding GOS to the ex vivo colonic system, preconditioned *L. reuteri* DSM 17938 significantly increased final lactate concentration compared to non-preconditioned *L. reuteri* DSM 17938 (17.5–18.3 mM with p = 0.016, respectively) and significantly increased final acetate concentration compared to adding only GOS (33.1–37.0 mM with p = 0.023, respectively). Concomitantly, this acid production also resulted in a more extensive pH decrease for the preconditioned strain compared to the non-preconditioned strain in the presence of GOS (pH decrease of 2.04 and 1.85, respectively). Overall, these metabolic results show that GOS presence is a necessary condition for preconditioning to result in a clear outcome (i.e., increase lactate and acetate production). In the next section, we look at the effects of GOS addition and preconditioning on microbial community dynamics. We detail a possible mechanism behind this increased lactate and acetate production and solidify this increase resulting from *L. reuteri* DSM 17938 and not from other members in the microbial community.

### Galacto-oligosaccharide addition affects overall microbial community composition, while preconditioning enhances engraftment of *L. reuteri* DSM 17938

**Figure 3:**
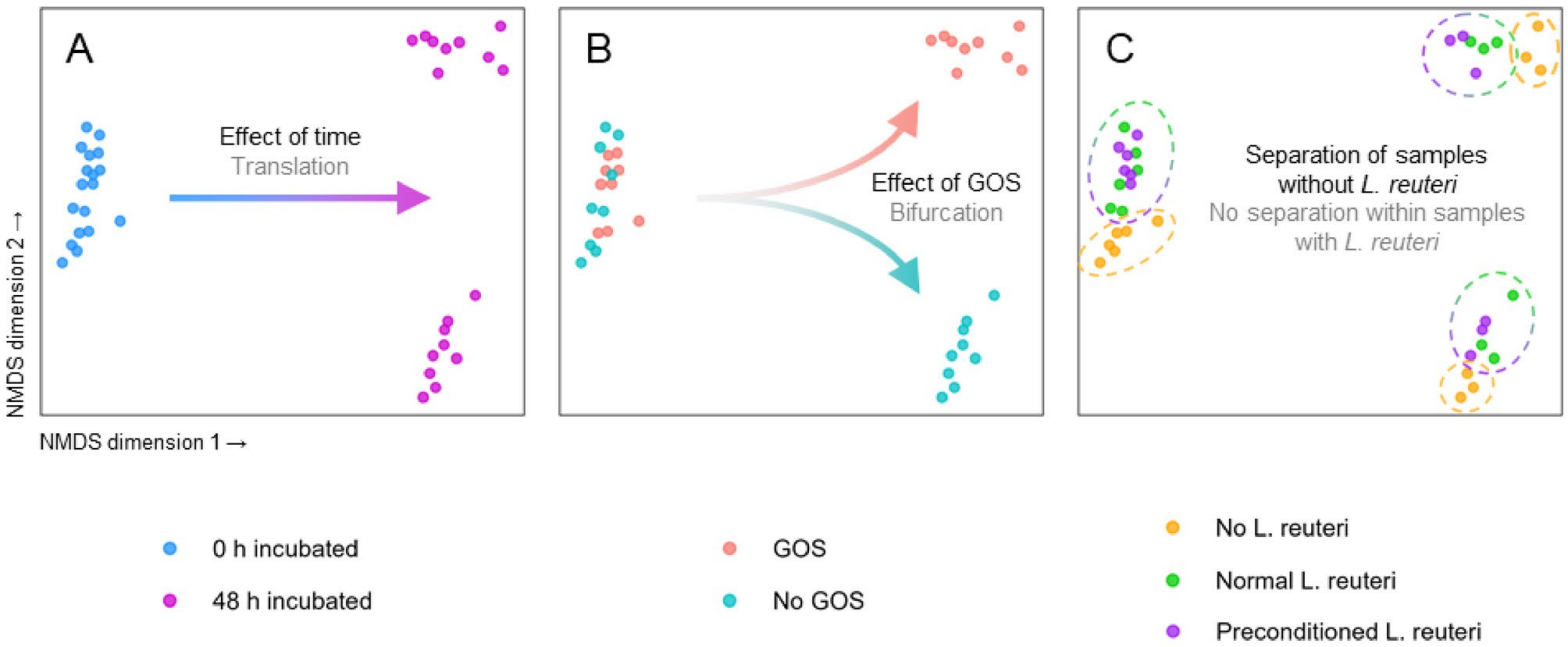
Microbial diversity in the SHIME ex vivo model displayed via non-metric multidimensional scaling shows how microbial dynamics are affected by incubation time, GOS, and preconditioning. Dots represent individual samples and are colored by different metadata attributes on each subplot A-C. Cluster separations were confirmed by permutational analysis of variance (PERMANOVA) at α = 0.05. Distance between dots represents the similarity of the underlying microbial community composition. Samples having a more similar microbial community composition are closer to each other and vice versa. (**A**) Diversity is shaped foremost by incubation time, as the distinct clusters are translated in the horizontal direction after 48 h. (**B**) For long incubation times, galacto-oligosaccharide (GOS) addition becomes a major factor determining microbial composition and divides communities that are associated with GOS addition and those who are not. Indeed, after 48 h incubation, clusters split solely based on GOS addition. A similar pattern is found for the microbial metabolite profiles (Fig. 2A). (**C**) Community composition is determined by *Limosilactobacillus reuteri* DSM 17938 addition but not by preconditioning. There are distinct clusters based on whether *L. reuteri* was added or not. This shows that *L. reuteri* DSM 17938 preconditioning does not affect the underlying microbial community composition differently from non-preconditioned *L. reuteri* DSM 17938.

**Figure 4:**
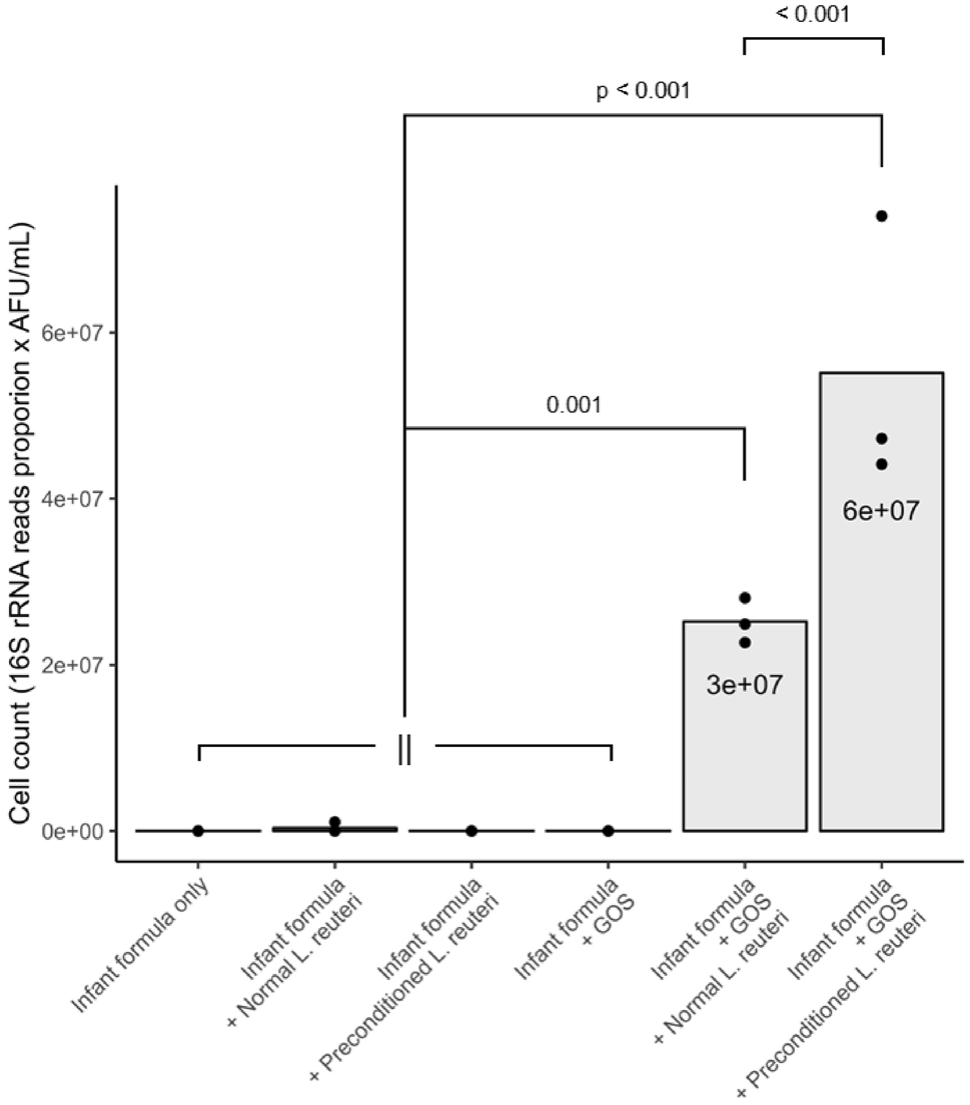
*Limosilactobacillus reuteri* levels after 48 h of ex vivo colonic incubation replicates (n = 3) are highest in presence of GOS. The taxon detected corresponds with high confidence to *Limosilactobacillus reuteri* DSM 17938 (see Sect. 2.2 “Galacto-oligosaccharide addition affects microbial overall community composition, while preconditioning enhances engraftment of L. reuteri DSM 17938” for a detailed explanation). The taxon *Limosilactobacillus reuteri* is not detected when *L. reuteri* DSM 17938 is not added (Infant formula only and Infant formula + GOS conditions), which validates the pipeline used. Relative abundances as determined by combining 16S rRNA sequencing and flow cytometry. *Limosilactobacillus reuteri* is detectable after 48 h only when adding galacto-oligosaccharides (GOS), showing that GOS addition enhances survival and successful engraftment of the strain. Preconditioning the strain results in higher abundances after 48 h, showing that preconditioning boosts engraftment when combined with GOS. Pairwise p-values are denoted with a bracket. All differences between normal *L. reuteri* DSM 17938 with GOS or preconditioned *L. reuteri* DSM 17938 with GOS and any other condition is significant. Notably, the preconditioned *L. reuteri* + GOS condition is significantly different from all other conditions, including the corresponding condition with non-preconditioned *L. reuteri* DSM 17938.

Permutational analysis of variance showed that incubation time, GOS, and *L. reuteri* DSM 17938 (both normal and preconditioned) meaningfully affected microbial community composition (Fig. 3). Apart from incubation time (Fig. 3A), GOS addition is the sole driver for the crisp composition bifurcation after 48 h of incubation (Fig. 3B). Notably, the taxon *L. reuteri* was only detected when *L. reuteri* DSM 17938 was added and was absent in the fecal inoculum (Supplementary Fig. S1). Additionally, amplicon sequence variants (ASVs) allocated to the taxon *L. reuteri* exhibit 100% nucleotide identity when aligned to the *L. reuteri* DSM 17938 reference genome (data not shown). These observations validate that these ASVs are highly likely to correspond to *L. reuteri* DSM 17938 experimentally supplemented. Bacterial communities like *L. reuteri*, *Bifidobacterium catenulatum*, *Bifidobacterium longum*, and *Escherichia coli* were associated with GOS addition. A bifidogenic effect was found for conditions with GOS, but this effect was somewhat reduced when *L. reuteri* DSM 17938 was added (Supplementary Fig. S1). In contrast to this, not adding GOS resulted in a more pronounced presence of *Faecalibacterium prausnitzii* and *Bacteroides fragilis*. This presence was accompanied by increased propionate and butyrate concentrations, two of the main metabolic end-products of these species. These results suggest that the availability of GOS represents a competitive advantage not only for bifidobacteria, but also for *L. reuteri* DSM 17938. Indeed, *L. reuteri* was detectable after 48 h of incubation only when adding GOS. Surprisingly, preconditioning *L. reuteri* DSM 17938 increased its final levels. When GOS was added, preconditioning resulted in significantly higher abundances of *L. reuteri* DSM 17938 compared to the non-preconditioned strain (6 × 10^7^ AFU/mL and 3 ×  10^7^ AFU/mL with p < 0.001, respectively; Fig. 4). Markedly, differences in community composition linked to addition of *L. reuteri* DSM 17938 resulted from the supplementation of the probiotic and not from preconditioning per se. Collectively, these observations corroborate the benefit of combining GOS and preconditioning *L. reuteri* DSM 17938 in a synbiotic formulation to establish the probiotic as a stable member of the microbial community (engraftment) during ex vivo colonic incubation. Because of the role of *L. reuteri* DSM 17938 and short-chain fatty acids (SCFAs) in bone health, we assessed how GOS and preconditioning affect osteoblastogenesis in the next section. Since probiotic engraftment was strongly linked to the presence of GOS, our osteal analyses focused on the 48 h samples in which GOS was added.

### Combining galacto-oligosaccharide and preconditioned *Limosilactobacillus reuteri* DSM 17938 enhances osteoblastogenesis

**Figure 5:**
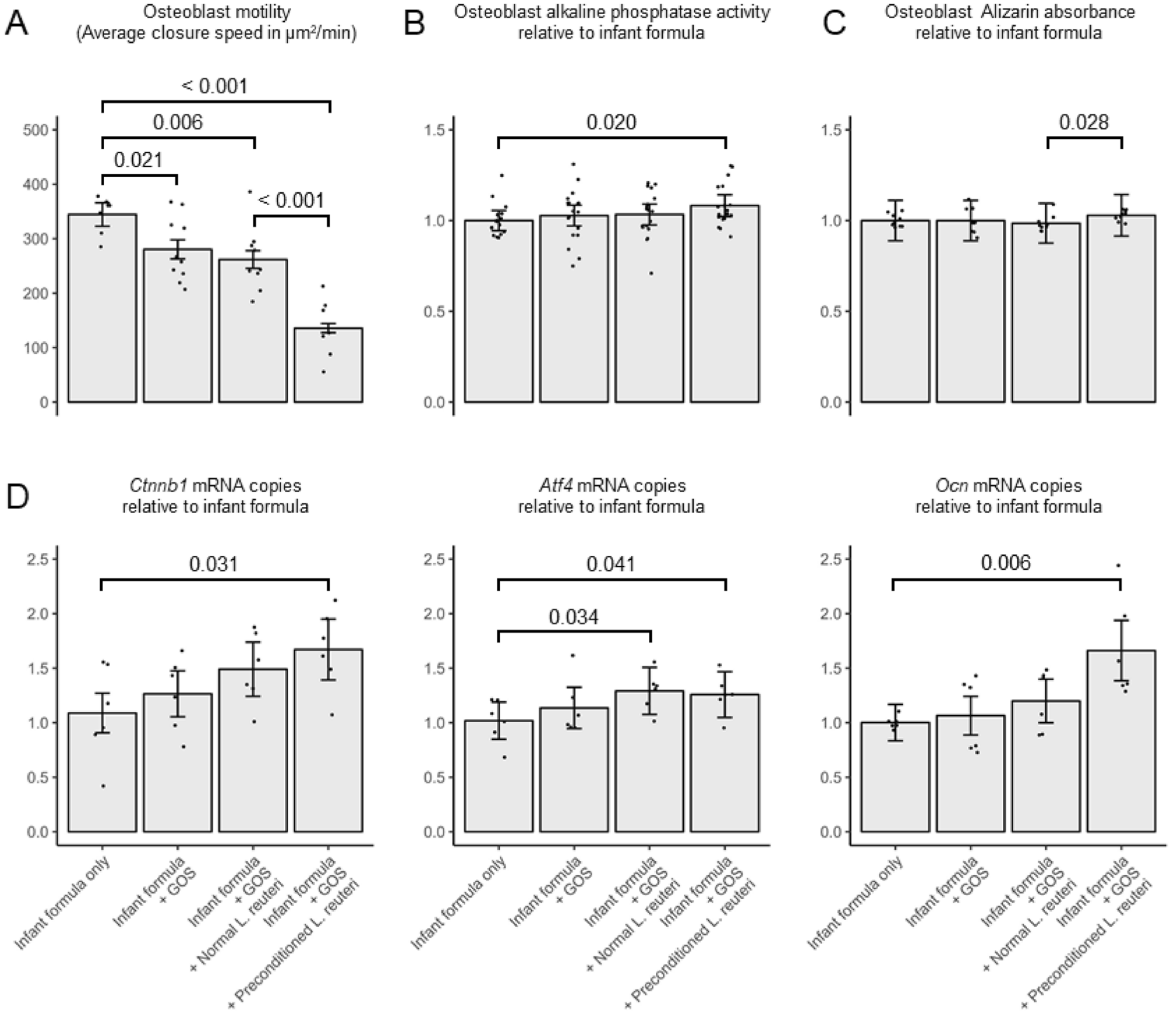
Galacto-oligosaccharide (GOS) addition and *Limosilactobacillus reuteri* DSM 17938 preconditioning enhance osteoblastogenesis. MC3T3-E1 cells were exposed to end products obtained after ex vivo colonic incubation. Error bars represent standard errors of replicate means. Pairwise p-values are denoted with a bracket. (**A**) Osteoblast motility assessed by the average speed of scratch closure assays (n = 16). (**B**) Osteoblast differentiation assessed by alkaline phosphatase activity normalized by infant formula condition (n = 18). (**C**) Osteoblast mineralization assessed Alizarin Red S optical density absorption (n = 9). (**D**) Osteoblast differentiation assessed by marker gene expression (*Ctnnb1*, *Atf4*, and *Ocn*) when exposed to short-chain fatty acids cocktails (n = 12). Cocktails mirror the ratios of acetate, propionate and butyrate obtained with the ex vivo colonic incubation.

Supernatants harvested from samples containing GOS or *L. reuteri* DSM 17938 did not significantly impact MC3T3-E1 osteoblast proliferation, which indicates the absence of cytotoxicity of these products (Supplementary Fig. S2). When combined with GOS, preconditioning *L. reuteri* DSM 17938 decreased osteoblast motility by 60%, 51% and 48% compared to infant formula only, GOS, or GOS combined with normal *L. reuteri* DSM 17938, respectively (Fig. 5A). This preconditioning-linked effect was translated into significantly higher alkaline phosphatase (ALP) activity compared to infant formula only (8% with p = 0.020, Fig. 5B). We were further able to confirm that preconditioned *L. reuteri* DSM 17938 combined with GOS significantly increased mineralization (4.5% with p = 0.028, Fig. 5C) compared to GOS with normal *L. reuteri* DSM 17938. Remarkably, GOS combined with non-preconditioned *L. reuteri* DSM 17938 did not result in any significant differences in ALP activity or mineralization. Furthermore, a cocktail of SCFAs mirroring the ratios in the samples coming from GOS combined with preconditioned *L. reuteri* DSM 17938 significantly increased key markers of osteoblast differentiation like activating transcription factor (*Atf4*), catenin beta 1 (*Ctnnb1*), and osteocalcin (*Ocn*) compared to infant formula by 25% (p = 0.041), 67% (p = 0.031), and 66% (p = 0.006), respectively (Fig. 5D). Like before, when *L. reuteri* DSM 17938 was not preconditioned, combination with GOS only increased ATF4 levels by 29% compared to infant formula. These analyses show that the increased SCFAs levels obtained when combining GOS with preconditioned *L. reuteri* DSM 17938 translate into increased osteoblastogenesis.

## Discussion

A probiotic-related benefit for the host is inherently linked to the strain’s presence in the gastro-intestinal tract. However, long-term engraftment is often challenging to achieve and difficult to predict from genomic data solely^34–36^. Consequently, ex vivo systems that mimic the gut environment provide a suitable means to study probiotic metabolite production and engraftment^36–38^. In this paper, we show that galacto-oligosaccharides (GOS) by itself and as a means for preconditioning *Limosilactobacillus reuteri* DSM 17938 have a measurable effect on ex vivo colonic metabolite profiles and microbial community dynamics. Microbial lactate and acetate production in the gut are beneficial for human health as they can be metabolized to other short-chain fatty acids (SCFAs) like butyrate and propionate. In general, SCFAs have been shown to provide a range of benefits including immune regulation, gastrointestinal, and osteal health^10,25,26,39^. Adding GOS in itself increases production of specific SCFAs, resulting in increased acidification (potentially due to background microbial community members), and can support *L. reuteri* DSM 17938 in engrafting in the microbial community. This suggests a competitive advantage for *L. reuteri* DSM 17938 in the gut ecosystem when using GOS as a carbon source and when having utilized a structurally similar carbon source during probiotic production^32,40^. Conversely, propionate producing members were diminished when supplementing GOS, potentially indicating their reduced competitiveness on this milk-derived substrate. This is substantiated by the increased final prevalence of *L. reuteri* DSM 17938 and putative β-galactosidase activity upon preconditioning the strain. This suggests that preconditioning-linked outcomes are unlikely to be the result of microbial community members besides *L. reuteri* DSM 17938. As such, this could mean that the higher *L. reuteri* DSM 17938 levels after 48 h of incubation compared to the non-preconditioned strain result from improved substrate competition for GOS when preconditioned. Indeed, compared to the non-preconditioned strain, preconditioning results in higher β-galactosidase activity as determined by colorimetric assay evaluating 5-bromo-4-chloro-3-indolyl β-D-galactopyranoside conversion rate (data not shown). We confirmed through testing of specific SCFAs cocktails that the ratios resulting from preconditioning *L. reuteri* DSM 17938 can at least partially explain the increased osteoblast differentiation we observed. Specific SCFAs like acetate, propionate, or butyrate indeed have a substantial effect on markers of osteoblast differentiation^10,25^. As acetate is one of the main metabolic end-products of *L. reuteri* DSM 17938, these observations suggest that GOS preconditioning-modulated SCFA production results in increased osteoblast differentiation indicators like motility and expression of key marker genes. Remarkably, exposing osteoblasts with the metabolic end-products of preconditioned *L. reuteri* DSM 17938 produced through ex vivo gastro-intestinal fermentation) also increases alkaline phosphatase activity and mineralization. An 8.0% increase in alkaline phosphatase activity and/or 4.3% increase in mineralization are in the same range of efficacy described for SCFAs in osteoblasts. Moreover, at the clinical level, an increase of 5% of bone mineral density, *i.e*., mineralization, can reduce fracture risk by 50%^42–45^. Previous studies showed positive effects of SCFAs on bone outcomes either by acting directly on cells such as osteoblasts, osteoclasts, and chondrocytes or indirectly by shaping an appropriate anti-inflammatory and immune regulatory response^46^. Two main mechanisms have been reported in the literature that may explain the effects on osteoblastogenesis; anti-inflammatory effects of gut metabolites or direct effect of SCFAs^39^. In our study, osteoblasts were directly stimulated through increased SCFA concentrations that directly act on differentiation through receptors expressed in mesenchymal stem cells. Specifically, G-protein-coupled receptors GPR41 and GPR43 can be activated by acetate, propionate, butyrate, or valerate, whereas GPR109a is mainly activated by butyrate^47^. Several studies have found that butyrate, at concentrations ranging between 500 nmol/L and 1 mmol/L, increases alkaline phosphatase production in murine calvarial organ cultures, *Runx2* transcription in MC3T3-E1 cells, and calcium content and osteoprotegerin expression of mineralized nodules in human osteoblasts^41,48^.

Increasing engraftment by adding GOS and preconditioning *L. reuteri* DSM 17938 shows the potential of a synergistic synbiotic rather than a complementary synbiotic, if evidence of selective utilization of the substrate were to be demonstrated in a study establishing a specific health benefit^49^. Complementary synbiotics consisting of *L. reuteri* and different carbohydrates have been evaluated in humans before^40^. In Rattanaprasert et al., even though *L. reuteri* DSM 17938 (not preconditioned in the latter study) was detectable in fecal samples, the addition of GOS did not increase fecal probiotic prevalence, nor was persistence enhanced after consumption had ended. Our results suggest that engraftment can be improved through preconditioning and that GOS is a required mediator. This increased engraftment could potentially be translated into a sustained health benefit like improvement of bone mineral density and reduction of fracture risk. Combining GOS and rhamnose was previously found to stimulate metabolic activity of *L. reuteri*^32,40^. We did not measure metabolic activity of *L. reuteri* directly, but we did follow general metabolic activity through short-chain fatty acid concentrations and acidification. From these measurements, an incremental increase in lactate and acetate concentrations was found when adding GOS. Still, it is possible that some fraction of lactate and acetate production is the result of members within the microbial community besides *L. reuteri* DSM 17938 since the ratio of acids cannot be explained by theoretical equimolar production through the phosphoketolase pathway. A substantial part of short-chain fatty acids is potentially produced by other highly prevalent members in the microbial community like *Escherichia coli* or *Bifidobacterium*, esp. considering the bifidogenic effect of GOS found in our study. This is apparent through the SCFA production in conditions in which GOS was supplemented without *L. reuteri* DSM 17938. The extent of the bifidogenic effect was halved when *L. reuteri* was supplemented. This mitigation suggests that *L. reuteri* DSM 17938 and bifidobacteria were competing for GOS as a growth substrate. As such, this suggests that the higher *L. reuteri* DSM 17938 levels after 48 h of incubation compared to the non-preconditioned strain result from improved substrate competition for GOS when preconditioning. Indeed, compared to the non-preconditioned strain, preconditioning results in higher β-galactosidase activity as determined by colorimetric assay evaluating 5-bromo-4-chloro-3-indolyl β-D-galactopyranoside conversion rate (data not shown).

Preconditioning with milk oligoscaccharides (MOS) has been implemented for *Bifidobacterium lactis* CNCM I-3446 in a similar ex vivo setting before^33^. In the latter study, the relative abundance of *B. lactis* increased when combined with MOS, suggesting a potential strong synbiotic effect of MOS on *B. lactis* engraftment. However, preconditioning *B. lactis* with MOS did not lead to improved engraftment, regardless of the presence of MOS. These findings are not entirely in line with our study, in which we found a benefit for engraftment not only by adding GOS, but also through preconditioning. To leverage the full benefit of preconditioning, the prebiotic moiety of a synergistic synbiotic formulation would perhaps need to be as structurally similar as possible to the preconditioning target compounds on top of the inherent ability of the probiotic to metabolize the prebiotic. Indeed, the latter study used MOS (consisting of galacto-oligosaccharides, lactose, and minor fractions of galactose and glucose) in its synbiotic formulation, while we used GOS (having a proportionally larger fraction of galacto-oligosaccharides)^50^. Alternatively, this might indicate that preconditioning-related benefits are simply species- or strain-specific. Nonetheless, our results show the utility of ex vivo trials to assess preconditioning-related benefits of synbiotic formulations, especially since the benefits might go beyond engraftment.

One limitation in our approach is that our results were generated using a relatively restricted analytical pipeline in a single donor biological triplicate ex vivo context and are consequently not suited to assess specific host interactions. Still, we believe that this study can be a steppingstone to larger-scale in vivo studies that would assess if our conclusions hold for larger donor groups in a human model^37,38^. We anticipate that these studies will provide further mechanistic understanding of our results.

## Conclusions

Galacto-oligosaccharides and *Limosilactobacillus reuteri* DSM 17938 modulate ex vivo colonic metabolite profiles and microbial community composition. Specifically, galacto-oligosaccharides increase short-chain fatty acid production and improve engraftment of *L. reuteri* DSM 17938 within a simulated colonic microbial community. Preconditioning *L. reuteri* DSM 17938 with GOS can further increase specific short-chain fatty production and enhance probiotic engraftment. Subsequent ex vivo testing of harvested supernatant from colonic incubation showed higher osteoblast differentiation and mineralization, two accepted surrogates of bone mineral density and bone strength. Increasing short-chain fatty acid production, engraftment, and bone formation potential by preconditioning when combined with galacto-oligosaccharides shows the superiority of this synergistic synbiotic formulation over a singular prebiotic or probiotic one to attain long-term engraftment in vivo. Although our conclusions will require confirmation in large-scale in vivo studies, preconditioning of *L. reuteri* DSM 17938 can potentially provide an efficient strategy to prevent age-related bone loss and limit fracture risk by optimizing probiotic metabolic activity and engraftment.

## Methods

### Medium components, carbohydrate mixtures, and infant formula

Unless mentioned differently, all medium components and reagents used were purchased from Merck KGaA (Darmstadt, Germany). The infant formula used was Lactogen Infant Formula (Société des Produits Nestlé S.A.; Vevey, Switzerland; Supplementary Table S1). The galacto-oligosaccharide mixture (GOS) used in these trials consisted of > 95% w/w galacto-oligosaccharides (≥ DP3) and < 5% w/w lactose and monosaccharides (New Franscisco Biotechnology Corporation Ltd.; Yunfu City; Candong, China). The milk-oligosaccharide mixture (MOS) used for probiotic production consisted of > 50% w/w galacto-oligosaccharides (≥ DP3), 30% w/w lactose, 10% w/w galactose and less than 10% w/w glucose and was produced with specifications as described before^50^.

### Bacterial strain and preconditioning

Commercial-grade *Limosilactobacillus reuteri* DSM 17938 culture powder was used for all ex vivo trials. To study the effect of GOS addition and strain preconditioning on GOS separately, two different processes were used to produce *L. reuteri* DSM 17938. In the first, *L. reuteri* DSM 17938 was grown in a standard industrial medium containing 8% glucose (w/w) and 8% fructose (w/w) as carbon sources and then spray dried (hereafter referred to as unconditioned *L. reuteri* DSM 17938). In the second, *L. reuteri* DSM 17938 was grown in the same growth medium, but with 6% MOS (w/w) and 6% fructose (w/w) as carbon sources before it was spray dried (hereafter referred to as preconditioned *L. reuteri*). MOS was used for preconditioning *L. reuteri* DSM 17938 due to its industrial relevance compared to GOS.

### Ex vivo* colonic incubations*

We implemented six different experimental conditions to study the effect of GOS addition and *L. reuteri* DSM 17938 preconditioning (Fig. 1). Biological triplicate short-term single-stage colonic simulator of the human intestinal microbial ecosystem (SHIME) experiments simulating the proximal large intestine were implemented as described before^38^. Infant formula was pre-digested to simulate gastric and small intestine conditions and then dialyzed through a cellulose membrane with a cut-off of 14 kDa prior to addition to the bioreactors as described before^51^. We used SHIME colonic background medium containing basal nutrients that are present in the colon (K_2_HPO_4_ 4.7 g/L; KH_2_PO_4_ 14.7 g/L; NaHCO_3_ 1.8 g/L; yeast extract 1.8 g/L; peptone 1.8 g/L; mucin 0.9 g/L; cysteine 0.5 g/L; polyoxyethylene sorbitan monooleate 20 1.8 mL/L) in all experiments. Depending on the specific experimental condition, GOS, unconditioned *L. reuteri*, or preconditioned *L. reuteri* DSM 17938 and infant formula were transferred to this basal medium at the start of the colonic incubations. A cryopreserved fecal sample of a 1.5-year-old toddler was used as inoculum. A stabilization regime for the fecal microbiota was implemented as described before^52^. Incubation vessels were kept under anaerobic atmosphere, stirred at 90 rpm, controlled at 37 °C, and ran for 48 h. When not processed immediately, samples were frozen at − 80 °C before analysis.

### Microbiological analyses

#### 16S rRNA gene amplicon sequencing, bioinformatic analysis, and flow cytometry

DNA of triplicate samples for each biological replicate was extracted as described before^53^. DNA extracts were sent out to LGC Genomics, GmbH (Teddington, UK) for 16S rRNA gene PCR targeting the V3-V4 region with the 341F (5′-CCTACGGGNGGCWGCAG-3′) and the modified 785R (5′-GACTACHVGGGTATCTAAKCC-3′) primers as described before^54^. Quality control of the PCR products was done using the Fermentas PCR Kit according to the manufacturer’s instructions (Thermo Fisher Scientific, Inc.; Waltham, Massachusetts, USA) and sequence length distribution was verified by electrophoresis on a 2% (w/v) agarose gel for 30 min at 100 V. The PCR products were sequenced on the 2 × 250 bp MiSeq platform (Illumina, Inc; San Diego, California, USA) according to the manufacturer’s standard protocol. Reads were converted into amplicon sequence variants (ASVs) using DADA2 version 1.15.2 with following filtering parameter values; truncLen = c(0,0), truncQ = 2, maxN = 0, and maxEE = c(2,2)^55^. Genus level taxonomy was assigned to ASVs using a naïve Bayesian classifier and the SILVA ribosomal RNA gene database version 132^56^. Species-level taxonomy was assigned using highest identity BLASTN hit in NCBI’s type material-restricted nucleotide collection database and cross-checked with the SILVA ribosomal RNA gene database where possible^57^. Total cell counts were determined using a CytoFLEX V2-B4-R2 flow cytometer (Beckman Coulter Life Sciences; Brea, California, US) with the PI/Syto 24 method as described in standard ISO 19344:2015^58^. The proportion of each ASV obtained through 16S rRNA gene amplicon sequencing was multiplied by the total cell counts obtained through flow cytometry to obtain absolute cell numbers for each ASV.

### Osteal analyses

#### MC3T3-E1 subclone 4 culture and treatments conditions

Since only the experimental conditions with GOS resulted in measurable *L. reuteri* DSM 17938 engraftment, only these conditions were selected for osteal analysis (with the infant formula only as a reference condition). The pre-osteoblastic cell line MC3T3-E1 subclone 4 (CRL-2593) was purchased from ATCC (Manassas; Virginia, USA). This cell line is described as a suitable in vitro model of osteoblast development that are key for bone mass acquisition during early life^24^. Cells were maintained in growth medium (GM) composed of ascorbic acid-free αMEM (ThermoFisher Scientific), supplemented with 10% fetal calf serum (FCS, ThermoFisher Scientific) and 1% penicillin/streptomycin. All culture media were refreshed every 2–3 days. Cells were passaged with trypsin/EDTA solution at less than 80% confluence. To induce differentiation into osteoblasts, cells were seeded at 5 × 10^4^ cells/cm^2^ and grown to confluency in GM for 24 h. Then, the medium was switched to differentiation medium (DM) composed of GM supplemented with 10 mM β-glycerophosphate and treatment solutions of interest. Cell culture treatments were performed in GM or DM containing 10% of sample or a cocktail of pure SCFAs. Samples were pre-processed by adjusting to pH 6.5 with NaOH, centrifuged at 4700 × g for 10 min, and supernatants filtered through 0.22 µm pore size membrane. The cocktail SCFA ratios mirrored sample SCFA concentrations: 22.98 mM acetate, 12.25 mM propionate, 3.63 mM butyrate for Infant formula only, 46.05 mM acetate, 14.20 mM propionate, 3.85 mM butyrate for Infant formula + GOS, 51.89 mM acetate, 13.42 mM propionate, 3.78 mM butyrate for Infant formula + GOS + Normal *L. reuteri*, and 56.60 mM acetate, 17.41 mM propionate, 4.56 mM butyrate for Infant formula + GOS + Preconditioned *L. reuteri*. These values were reduced by a factor 1000 to reach relevant physiological levels.

#### Short-chain fatty acids concentrations analysis and cocktail preparation

Short and branched chain fatty acids (acetate, propionate, butyrate, valerate, iso-butyrate and iso-valerate) were quantified using a solid phase microextraction gas chromatography coupled to mass spectrometry (SPME–GC–MS, Agilent; Santa Clara, California, USA) as described previously with minor modifications^59^. Briefly, 50 µL of samples were stabilized with 0.5% ortho-phosphoric acid, labeled internal standards (^2^H_3_-acetic acid, ^2^H_5_-propionic acid, ^2^H_5_-butyric acid, ^2^H_7_-isobutyric acid, ^2^H_9_-valeric acid, and ^2^H_9_-isovaleric acid) were added and vortexed gently. The SPME–GC–MS conditions were as follows: PDMS/DVB fiber (Supelco; Bellefonte, Pennsylvania, USA), agitation at 40 °C with an extraction time of 10 min. Temperature and time of desorption were set at 250 °C for 5 min. The GC conditions were as follows: inlet temperature at 250 °C with as carrier gas. The temperature program was 100 °C for 4 min, followed by an increase to 240 °C at 11 °C/min for a total time of approx. 16.7 min. Calibration curves were made by plotting ratio between each metabolite and their respective internal standard peak areas against the theoretical standard concentration. The concentrations were then determined with a linear regression model with a weighted 1/x model. Repeatability and intermediate reproducibility (expressed as coefficient of variation) were lower than 15% for all the metabolites measured.

#### Osteoblast proliferation

MC3T3-E1 subclone 4 cells were seeded in GM on a 96-well plate at a density of 320 cells/well. After 24 h, treatment compounds of interest were added (day 0) and the plate was transferred in the IncuCyte ZOOM live cell imaging system (Essen BioScience; Ann Arbor, Michigan, USA) for 4 days. Fresh culture medium was added after 2 days. The confluence percentage was evaluated at day 4 and was normalized to the corresponding wells at day 0. These values were then re-normalized to the control values (infant formula only or SCFA cocktail condition, depending on the experiment).

#### Osteoblast motility

MC3T3-E1 subclone 4 cell migration assays were performed using Culture-Insert 2 wells (Ibidi; Gräfelfing, Germany) according to the manufacturer’s instructions. Cells were seeded at 10 × 10^5^ cells/cm^2^. After 24 h, the supernatant and Culture-Inserts were removed. Cells were incubated with GM containing only 2% FBS and supplemented with 1% sample or SCFA cocktail. Cells colonization of the “wound” was monitored by the IncuCyte ZOOM live cell imaging system (Essen BioScience) in increments of 2 h. The wound healing closure speed was analyzed with FastTrack AI (Ibidi).

#### Osteoblast alkaline phosphatase activity

MC3T3-E1 subclone 4 cells were differentiated for 7 days and were then collected for alkaline phosphatase (ALP) activity measurement as described previously with minor modifications^60^. Briefly, cells were lysed by heat shock and collected in ALP buffer (1 M diethanolamine, 0.24 M MgCl_2_, pH 9.8). Enzymatic reaction was monitored at 405 nm after 4-Nitrophenyl phosphate disodium salt hexahydrate addition. Michaelis–Menten kinetics were evaluated at 30 °C for 30 min. V_max_ was used as a proxy for ALP activity. The activity value was normalized by protein content measured via the Pierce BCA Protein Assay Kit (ThermoFisher Scientific) according to the manufacturer’s instructions.

#### Osteoblast Alizarin Red S absorbance

MC3T3-E1 subclone 4 cells were differentiated for 21 days and were then collected for Alizarin Red S staining as described previously with minor modifications^61^. Briefly, cells were fixed twice with 70% ethanol for 15 min. Cells were washed with reverse osmosis filtered water and stained with 40 mM Alizarin Red S (3,4-Dihydroxy-9,10-dioxo-9,10-dihydroanthracene-2-sulfonic acid) solution for 30 min (ThermoFisher). Stained cells were washed multiple times with reverse osmosis filtered water and PBS. Plates were then dried. The dye was dissolved in 10% cethylpyridinium chloride solution for 15 min and the absorbance measured at 562 nm.

#### RNA extraction

MC3T3-E1 subclone 4 cells were differentiated for 7 or 21 days and were then collected for gene expression analysis. RNA was extracted using the RNeasy plus mini kit (Qiagen; Hilden, Germany) with the QIAcube (Qiagen,) according to the manufacturer’s instructions. Briefly, cells were lysed in RLT buffer and spun in a QIAshredder column (Qiagen) before being processed by the QIAcube. RNA concentrations were measured using the DropSense96 (TRINEAN, Gentbrugge, Belgium).

#### Reverse transcription and quantitative PCR (qPCR)

cDNAs were synthetized using qScript cDNA SuperMix (Quantabio; Beverly, Massachusetts, USA) according to the manufacturer’s instructions. Briefly, 0.9 µg of RNA mixed to SuperMix and RNase-free water were reverse transcribed with the following program: 5 min at 25 °C, 30 min at 42 °C, and 5 min at 85 °C. cDNAs were diluted 9X with RNase-free water and used for qPCR using the LightCycler 1536 DNA Green Master kit (Roche; Basel, Switzerland) according to the manufacturer’s instructions. Briefly, cDNAs were diluted 7X in a solution containing the master mix, the Bright Green, and the DNA primers targeting activating transcription factor 4 (*Atf4*), catenin beta 1 (*Ctnnb1*), and osteocalcin (*Ocn*) with following nucleotide sequences *Atf4*-f GGCAAGGAGGATGCCTTTT, *Atf4*-r CGAAACAGAGCATCGAAGTCA; *Ctnnb1*-f TGCCTTCAGATCTTAGCTTATGG, *Ctnnb1*-r AGACAGCACCTTCAGCAC; *Ocn*-f ACCATCTTTCTGCTCACTCTG, *Ocn*-r GTTCACTACCTTATTGCCCTCC. Reaction ran in a LightCycler 480 II (Roche) with the following program: 7 min at 95 °C, 40 cycles of 1 s at 95 °C and 30 s at 60 °C. The relative gene expression was evaluated via the 2^-ΔΔCt^ method.

### Statistical analyses

Where not explicitly stated, statistics were calculated using R version 3.6.3 base functions. Relevant assumptions for statistical testing were checked beforehand and tests were implemented only when assumptions were unviolated. Unless stated otherwise, reported values are averages of biological triplicates. Differences were assessed by two-sided Student's t-test and considered at significance level α = 0.05 (H_0_: true difference = 0 and H_A_: true difference ≠ 0) and p-values explicitly expressed up to 0.001 for conciseness (after which they are simply denoted as p < 0.001). When making multiple comparisons, p-values were corrected with the Benjamin-Hochberg method. Beta diversity of microbial communities was assessed with non-metric multidimensional scaling and permutational analysis of variance with 10^5^ permutations using vegan version 2.5–6^62^. PERMANOVA results were considered at significance level α = 0.05 (H_0_: group centroids and dispersion equivalent in measure space and H_A_: group centroids and dispersion not equivalent in measure space). For experiments with MC3T3-E1 cells, analysis was performed with Prism 8 (GraphPad; San Diego, California, USA). Pairwise comparisons were made via the Mann–Whitney test at significance level α = 0.05 (H_0_: true difference = 0 and H_A_: true difference ≠ 0).

### Supplementary Information

Supplementary Information.

## Data Availability

The datasets generated during and/or analysed in the current study are available from the corresponding author on reasonable request. The 16S rRNA seq data used in this study have been deposited in the European Nucleotide Archive under accession numbers ERR11701890–ERR11701925 in project PRJEB64270.
