# Supplementary material for: Galacto-oligosaccharide preconditioning improves metabolic activity and engraftment of Limosilactobacillus reuteri and stimulates osteoblastogenesis ex vivo
Source: Sci Rep. 2024 Feb 21;14:4329. doi: 10.1038/s41598-024-54887-z (PMC10881571; doi:10.1038/s41598-024-54887-z)
Supplement: Supplementary file 1 — Supplementary Information. [file 41598_2024_54887_MOESM1_ESM.pdf]

Supplementary material for Galacto-oligosaccharide preconditioning improves metabolic activity and engraftment of *Limosilactobacillus reuteri* and stimulates osteoblastogenesis *ex vivo*

Title of manuscript: Galacto-oligosaccharide preconditioning improves metabolic activity and engraftment of Limosilactobacillus reuteri and stimulates osteoblastogenesis *ex vivo*

Author list: Florac De Bruyn, Nicolas Bonnet, Michaël Baruchet, Magalie Sabatier, Isabelle Breton, Bertrand Bourqui, Ivana Jankovic, Marie-Noëlle Horcajada, Guénolée Prioult

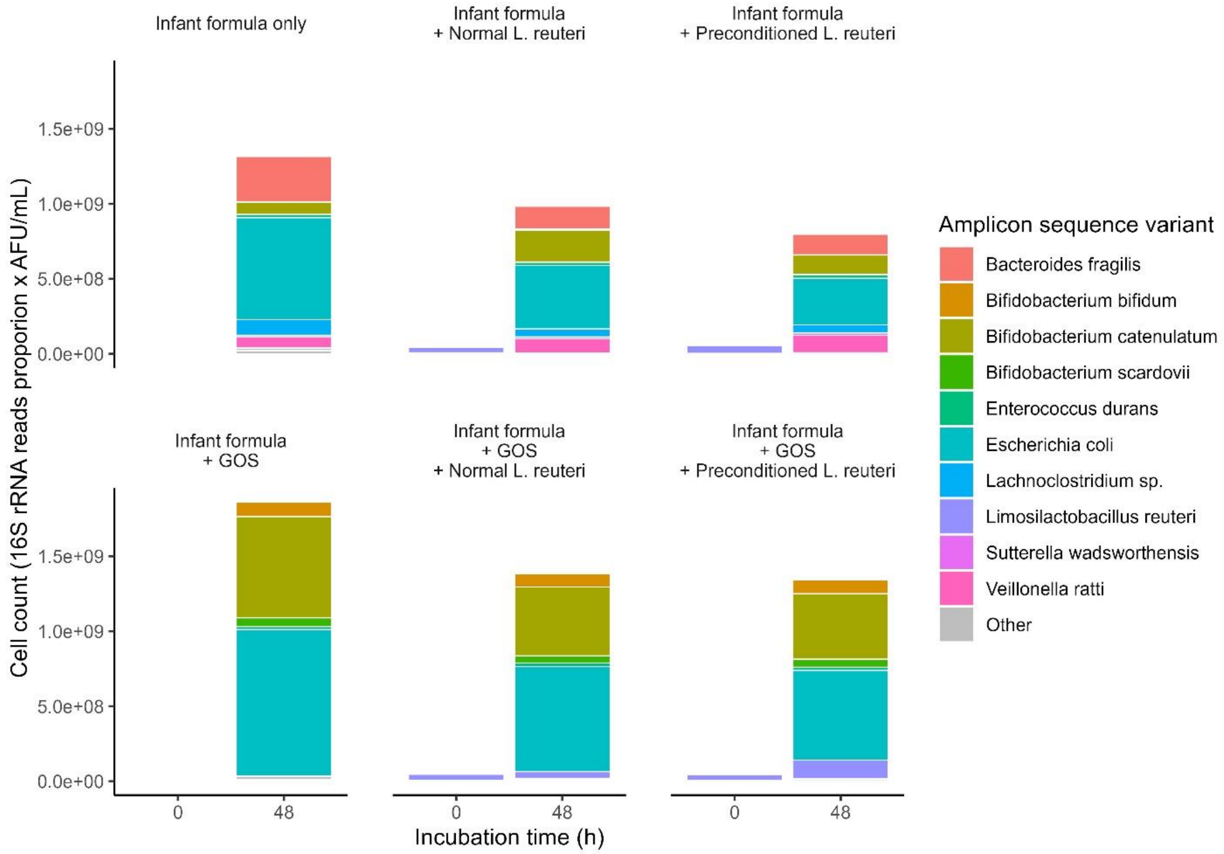

Supplementary Figure S1. Changes in bacterial community composition during colonic incubation. The 10 most abundant species are shown (representing 92% of all reads). Overall, *Escherichia coli*, *Bifidobacterium catenulatum*, and *Bacteroides fragilis* are prominent members. *Limosilactobacillus reuteri* is detectable when supplemented. Bifidobacteria are more prevalent when galacto-oligosaccharides are supplemented.

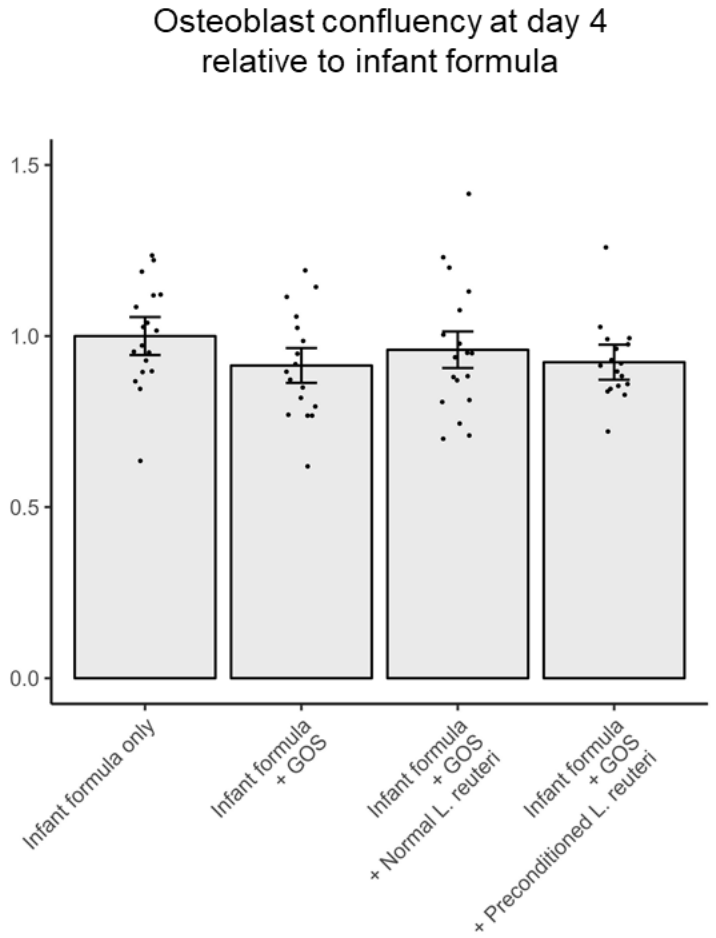

Supplementary Figure S2. Galacto-oligosaccharide (GOS) addition and *Limosilactobacillus reuteri* DSM 17938 preconditioning do not affect osteoblast proliferation. MC3T3-E1 cells show no significant differences in confluency after 4 days of exposure to end products obtained after *ex vivo* colonic incubation. Error bars represent standard error of replicate means (n = 18).

Supplementary Table S1: Nutritional composition of the milk matrix used in this study.

Parameter	Value	Unit
Total water	2.86	g/100 g
Total fat	23.15	g/100 g
Total nitrogen	1709.07	mg/100 g
Total protein	10.68	g/100 g
Calcium	477	mg/100 g
Magnesium	67.70	mg/100 g
Sodium	257	mg/100 g
Potassium	642	mg/100 g
Chloride	324	mg/100 g
Phosphorous	299	mg/100 g

Supplementary Table S2. BLASTN best hits for 10 largest amplicon sequence variants used for species-level taxonomy allocation.

Amplicon sequence variant number ordered by total parent reads	BLASTN best hit	Sequence accession number
1	*Escherichia coli*	MN307293.1
2	*Bifidobacterium catenulatum*	LC483552.1
3	*Limosilactobacillus reuteri*	MN865144.1
4	*Bacteroides fragilis*	CP036555.1
5	*Faecalibacterium prausnitzii*	NR_028961.1
6	*Bifidobacterium longum*	LR134369.1
7	*Veillonella ratti*	NR_113377.1
8	*Bifidobacterium bifidum*	LR134344.1
9	*Enterococcus durans*	MK330566.1
10	*Lachnoclostridium edouardi*	LT671595.1
